# Impact of Diagnostic and Treatment Delays on Survival and Treatment-Related Toxicities in Portuguese Patients With Head and Neck Cancer

**DOI:** 10.7759/cureus.53039

**Published:** 2024-01-27

**Authors:** Bárbara Machado, Tiago Barroso, Joana Godinho

**Affiliations:** 1 Medical Oncology, Centro Hospitalar de Entre Douro e Vouga, Santa Maria da Feira, PRT; 2 Medical Oncology, Centro Hospitalar Universitário Lisboa Norte, Lisbon, PRT

**Keywords:** covid-19, risk factors, treatment toxicity, survival, time until treatment initiation, time to diagnosis, head and neck cancer

## Abstract

Introduction: Delays in diagnosis and initiation of treatment have a negative impact on the prognosis and survival of head and neck cancer (HNC) patients. These delays also involve more intensive treatments with greater toxicity, dysfunction, and morbidity.

Methods: This was a retrospective observational study with patients diagnosed with HNC between January 1, 2018, and December 31, 2021. The main objective was to estimate whether the time to diagnosis (TD) and time until treatment initiation (TIT) translated into changes in the patient's overall survival (OS). Multivariate data analysis was performed with the Cox regression model. Significance was considered for p<0.05.

Results: A total of 139 patients were included in this study. Median TD was 126 days and median TIT was 43 days. No association between TD, TIT, treatment toxicity, and OS was found. Being a smoker was associated with a longer TD (p=0.05, hazard ratios {HR}=1.01). TIT was significantly shorter in higher grades (p=0.03, HR=0.57) and during coronavirus disease 2019 (COVID-19) (p=0.04, HR=0.57), but higher in larger disease (tumor {T}) (p=0.04, HR=1.39). A higher T (p=0.01, HR=2.67) and lymph node metastasis (nodes {N}) (p=0.02, HR=2.24) were identified as risk factors with a negative impact on OS, whereas grade was positively correlated (p=0.05, HR=0.32).

Conclusions: Even though there was no correlation between TD and TIT, and OS, action still needs to be taken to shorten these times. T and N remain negative predictive prognostic markers of HNC.

## Introduction

Head and neck cancer (HNC) includes malignant tumors that develop in the oral cavity, pharynx, larynx, sinuses and paranasal glands, and salivary glands [1-4]. Squamous cell carcinoma of the head and neck (HNSCC) is the most common histological type and is described as the sixth or seventh most frequent worldwide [3,5]. In 2020, 931,931 and 2,658 new cases of HNC were diagnosed worldwide and in Portugal, respectively [6,7]. In turn, in 2020, this cancer was responsible for 467,125 deaths worldwide, constituting the sixth cause of death from cancer with 1,227 deaths in Portugal [6,7]. HNC is associated with a high mortality and morbidity rate [8], and survival remains poor, despite advances in treatment modalities [9]. The main prognostic factor is the stage of the disease [9,10], and most tumors are asymptomatic until more advanced stages [5] or cause symptoms and/or signs often confused with benign situations [4,10]. Also, there is no effective screening method to detect head and neck cancers (HNCs) at early stages [5,10]. According to the literature, delays in diagnosis and initiation of treatment have a negative impact on the prognosis and survival of patients [10-13]. There are some causes described for diagnosis delay, such as sociocultural and behavioral factors like smoking and/or alcohol habits, beliefs, and/or devaluation or normalization of signs and symptoms [8,9], which go unnoticed by physicians with little experience in HNC [4,9]. The evolution of imaging methods and treatments and the consequent increasing complexity of the diagnostic and therapeutic approach may lead to an increase in the time to initiation of treatment (TIT) [12]. Other proposed causes for delays in the TIT are related to purely administrative factors [11]. The authors, Murphy et al. demonstrated that, when this interval is greater than 46-52 days, there is an impact on mortality, particularly in the early stages (I and II) [12]. These delays also involve more intensive treatments with greater toxicity, dysfunction, and morbidity [11,13]. There is still insufficient evidence to associate delays in diagnosis and treatment initiation with the long-term effects in terms of toxicity and dysfunction reported by the patient.

Therefore, this study aimed to estimate the time until diagnosis and the initiation of treatment in a Portuguese head and neck cancer population and verify whether these factors have an impact on survival and treatment-related toxicities. Also, we wanted to understand the reasons related to these delays and if the coronavirus disease 2019 (COVID-19) pandemic had an influence on the time until diagnosis, treatment beginning, and survival.

## Materials and methods

This was a retrospective observational study with the collection of clinical data from patients diagnosed with head and neck cancer between January 1, 2018, and December 31, 2021, at the Centro Hospitalar de Entre Douro e Vouga (CHEDV) in Portugal. The data were collected, until August 31, 2023, from the clinical files of the patients and recorded in an Excel sheet created exclusively for this purpose, with restricted access to the researchers. For the protection of personal data, an identification code was assigned to each patient, maintaining anonymity in the database. A data collection form was developed to fill in with demographic data, tumor data, dates of diagnosis and initiation of treatment, treatment, and survival data.

Patients were included if they were aged 18 years or older and had a new diagnosis of histologically proven head and neck cancer. Exclusion criteria included a previous diagnosis of oncological disease, thyroid and skin tumors, melanomas, and HNC with exclusively neuroendocrine or hematological histology.

The primary endpoint was to estimate whether the time until diagnosis and the beginning of treatment translated into changes in the patient's survival. Secondary endpoints included the following: assessing whether the interval between diagnosis and treatment initiation had an impact on treatment toxicity; estimating whether there was a difference in the time to diagnosis and initiation of treatment during the COVID-19 pandemic and whether it had an impact on survival; determining if there were population- and/or disease-related risk factors that caused a delay in the time to diagnosis and the time until treatment initiation.

The time to diagnosis (TD) was defined as the time from the appearance of the first symptom(s) to the histological diagnosis of head and neck cancer. Time until treatment initiation (TIT) was defined as the time from diagnosis until the beginning of treatment. We also used other intervals in our analysis, such as patient’s time (PT) (the time from the appearance of the first symptom{s} to the first contact with a health institution) and the specialty time (ST) (the time from the appearance of the first symptom{s} to observation by a medical oncologist, surgeon or otorhinolaryngologist). Time was measured in days and was accessed as a continuous variable. The analysis of the impact of the COVID-19 pandemic was conducted between March 10, 2020, the date on which the World Health Organization (WHO) classified COVID-19 as a pandemic, and December 21, 2021. Demographic data included factors related to the patients, such as gender, age, Eastern Cooperative Oncology Group Performance Status (ECOG PS) at diagnosis, smoking status, alcohol habits, residence, employment status, and tumor-related factors, including site, histology, tumor-nodes-metastasis (TNM) classification, stage, and grade. A maximum of two initial symptoms were recorded for each patient. Overall survival was measured from the time of diagnosis until the event of death or last visit to the hospital. Diagnosis and staging were performed based on the American Joint Committee on Cancer (AJCC) eighth edition. Treatment toxicities were reported according to Common Terminology Criteria for Adverse Events (CTCAE) version 5. G3 and/or G4 toxicity, dose reductions, or treatment discontinuations due to toxicity were classified as high-grade toxicity. The impact of treatment toxicity in TD and TIT was estimated using this measure.

Statistical analysis was performed with a custom program written in the Python programming language [14]. For survival analysis, we used the Lifelines Python package, and for plots, we used the Matplotlib package [15,16]. Descriptive statistics and time delays were presented as means, medians, standard deviations, frequencies, and proportions, accordingly. Survival estimates and time delays were calculated with Kaplan-Meier method. The impact of predictor variables on the time delays and overall survival was assessed using a multivariate Cox regression. While in the overall survival analysis, there are censored data that correspond to patients for whom the time of death was not yet observed, in the time delays there is no censored data by design, as all included patients had a diagnosis and had started treatment at the time of data collection.

The Cox regressions report the coefficient values for each variable and their respective hazard ratios (HR). For the Cox regression, no transformations were required for continuous variables. Binary variables were dichotomized into binary 0 or 1 values (as in the case of smoker vs no smoker, rural vs urban, among others). Tumor location (oral cavity, pharynx, hypopharynx, and larynx) and tumor prognostic group (stage I, II, III, and IV) were dichotomized. Patients were divided into oral cavity vs no oral cavity, pharynx vs no pharynx, stage I vs not stage I, and similarly for the other variables. The variables tumor grade, tumor stage T, and tumor stage N were converted into integers from 1 to 3 (corresponding to grade 1, 2, or 3), 1 to 4 (corresponding to T1, T2, T3, T4). The tumor stage metastasis (M) is naturally binary and was encoded as 0 or 1. We considered coefficients with a p<0.05 as significant. No adjustment was made for multiple comparisons.

## Results

Of the 160 patients initially included, 21 were excluded because they did not meet the following inclusion criteria: HNC recurrence (n=7, 33%), previously diagnosed tumors unrelated to HNC (n=7, 33%), and diagnoses of other cancers besides HNC (thyroid and skin carcinoma) (n=7, 33%). A descriptive analysis of characteristics of patients and their tumors was performed on 139 patients in total (Table 1). Median age was 63 (min: 32 - max: 92) years. The majority of patients had an ECOG PS 1 (n=96, 69.1%) and were male (n=117, 84.2%). The majority (n=78, 56.1%) were current smokers with a median of 40 pack-years and had moderate/excessive alcohol intake habits (n=72, 51.8%). The most frequent histology was squamous cell carcinoma (SCC) (n=126, 90.6%), with the larynx (n=47, 33.8%) and oropharynx (n=40, 28.8%) being the most common sites. The majority of patients had advanced disease at diagnosis; the most prevalent presentations were T4 (n=43, 31.9%), T3 (n=42, 31.1%), and N2 (n=40, 29.2%). Most patients presented with at least one symptom (n=135, 97%), dysphonia (n=52, 37.4), odynophagia (n=35, 25.2%), and a lump in the neck (31, 23.5%) the most prevalent ones. Appendix 1 represents the symptoms of HNC patients. Concerning the search for health care, emergency service (ES) was the main place for first medical observation (n=59, 42%), followed by primary care physician (n=52, 37%) with only two patients going to the dentist. Nevertheless, 12% (n=16) of the patients' first medical contact was an otolaryngologist (ENT) consultation in a private hospital. Regarding first specialty contact, an ENT was put forward for nearly all patients (n=121, 87%). The median time until this consultation was 94.9 days if the patient was first observed in the ES, compared with median 208 days after primary care physician consultation. The majority of patients were diagnosed with HNC during hospitalization (n=72, 52%) (Appendix 2).

**Table 1:** Characteristics of patients and their tumors. ECOG PS: Eastern Cooperative Oncology Group Performance Status; n: number of patients; SCC: squamous cell carcinoma; TNM: tumor-nodes-metastasis; Tx: primary tumor can not be assessed

Patient’s and tumor’s factors	n (%)
Age (years)	Median: 63 (min: 32, max: 92)
Gender	
Male	117 (84.2)
Female	22 (15.8)
ECOG PS	
0	16 (19.3)
1	96 (69.1)
2	11 (7.9)
3	14 (10.1)
4	4 (2)
Employment status	
Currently employed	43 (30.9)
Unemployed	23 (16.5)
Retired	63 (45.3)
Missing	10 (7.2)
Localization	
Rural	76 (54.7)
Others	63 (45.3)
Tobacco history	
Current smoker	78 (56.1)
Former smoker	27 (19.4)
Never smoker	30 (21.6)
Missing	4 (2.9)
Excessive use of alcohol	
Yes	72 (51.8)
Previous	24 (17.3)
No	39 (28.1)
Missing	4 (2.9)
Site	15 (10.8)
Oral cavity	40 (28.8)
Oropharynx	18 (12.2)
Hypopharynx	7 (5)
Nasopharynx	47 (33.8)
Larynx	9 (6.5)
Nose and paranasal sinus	2 (1.4)
Major salivary glands	2 (1.4)
Histology	
SCC	126 (90.6)
Adenocarcinoma	8 (5.8)
Others	5 (3.6)
Tumor (TNM)	
Tx	2 (1.4)
1	26 (19.3)
2	24 (17.8)
3	42 (31.1)
4	43 (31.9)
Missing	2 (1.4)
Node (TNM)	
0	63 (46)
1	15 (10.9)
2	40 (29.2)
3	19 (13.9)
Missing	2 (1.4)
Metastasis (TNM)	
0	129 (93.5)
1	9 (6.5)
Stage	
I	24 (17.3)
II	15 (10.8)
III	27 (19.4)
IV	72 (51.8)
Missing	1 (0.7)
Grade	
1	86 (62)
2	21 (15)
3	12 (9)
4	7 (5)
Missing	13 (9)

Regarding the study's main objective, median overall survival (OS) was not reached, but at the end of the study, 46% (n=64) of patients had died. Medians and quartiles (Q1-Q3) for time to diagnosis (TD) were 126 (60-260) days and for the time until treatment initiation (TIT) were 41.5 (7-66) days. TD and TIT were not correlated with the patient’s survival (p=0.08 and p=0.18, respectively) (Table 2). Nevertheless, 18% (n=25) of patients didn’t get any antineoplastic treatment because of their poor functional status after diagnosis and passed away before they were able to start treatment. Although this was not the objective of this study, an additional investigation revealed that the quartiles and medians (Q1-Q3) for PT were 52 (10-95) days, but it had no effect impact on survival (p=0.51) and median ST was 77 (31 {Q1} - 172 {Q3}) days (Table 2).

**Table 2:** Times and tumor and patients’ factors and survival multivariate analysis. *log_2_(p): 4.47 P-value <0.05 was considered statistically significant. TD: time to diagnosis; TIT: time until treatment initiation; PT: patient's time; HR: hazard ratio; CI: confidence intervals; COVID-19: coronavirus disease 2019; TNM: tumor-nodes-metastasis; ECOG PS: Eastern Cooperative Oncology Group Performance Status

Times and factors	p-Value	HR (95% CI lower-upper)
TD	0.08	-
TIT	0.18	-
PT	0.28	-
COVID-19	0.51	-
High-grade toxicity	0.80	-
Tumor (TNM)	0.01	2.67 (1.26-5.67)
Node (TNM)	0.02	2.24 (1.12-4.46)
Metastasis (TNM)	0.12	-
Grade	0.05	0.32 (0.10-0.98)*
Oral cavity	0.48	-
Oropharynx	0.89	-
Nasopharynx	0.95	-
Larynx	0.95	-
Others	0.90	-
Sex	0.85	-
ECOG PS	0.34	-
Tobacco	0.08	-
Alcohol	0.64	-
Employed	0.13	-
Rural	0.19	-

Regarding treatments and treatment-related toxicities, curative intent treatment was the most prevalent among the patients who were able to start therapy (n=100, 87.8%). The most common treatments were surgery alone (n=26, 22.8%), chemoradiotherapy/radiotherapy (ChT/RT) (n=25, 21.9%), and surgery plus adjuvant radiation (RT) (n=21, 18.4%). Toxicities G3 or G4 were reported in 17.3% (n=249) of patients. Nine patients (n=7.9%) had to stop their therapy because of toxicity, and four patients (n=3.5%) required to lower the dosage. Appendix 3 provides more information concerning toxicity. High-grade toxicity impacted neither TD (p=0.10), TIT (p=0.59), PT (p=0.69), ST (p=0.70) nor survival (p=0.8) (Tables 2-4).

**Table 3:** TD, TIT, and tumor and patients’ factors multivariate analysis. *log_2_(p): 4.34 P-value <0.05 was considered statistically significant. TD: time to diagnosis; TIT: time until treatment initiation; Q1: first quartile; Q3: third quartile; HR: hazard ratios; CI: confidence intervals; COVID-19: coronavirus disease 2019; ECOG PS: Eastern Cooperative Oncology Group Performance Status

TD			TIT	
Factors	p-Value	HR (95% CI lower-upper)	p-Value	HR (95% CI lower-upper)
COVID-19	0.31	-	0.04	0.57 (0.34-0.96)
High-grade toxicity	0.10	-	0.59	-
Tumor (TNM)	0.87	-	0.04	1.39 (1.01-1.92)
Node (TNM)	0.75	-	0.75	-
Metastasis (TNM)	0.30	-	0.19	-
Grade	0.68	-	0.03	0.57 (0.33-0.96)
Oral cavity	0.43	-	0.95	-
Oropharynx	0.94	-	0.76	-
Nasopharynx	0.88	-	0.73	-
Larynx	0.88	-	0.73	-
Others	0.95	-	0.87	-
Sex	0.43	-	0.61	-
ECOG PS	0.97	-	0.92	-
Tobacco	0.01	1.01 (1.00-1.02)*	0.11	-
Alcohol	0.51	-	0.14	-
Employed	0.30	-	0.26	-
Rural	0.51	-	0.61	-

**Table 4:** PT, ST, and tumor and patients’ factors multivariate analysis. PT: patient's time; ST: specialty time; COVID-19: coronavirus disease 2019; ECOG PS: Eastern Cooperative Oncology Group Performance Status

PT			ST		
Factors	p-Value	HR (95% CI lower-upper)	p-Value		HR (95% CI lower-upper)
COVID-19	0.86	-	0.70	-	
High-grade toxicity	0.69	-	0.70	-	
Tumor (TNM)	0.04	0.70 (0.50-0.99)	0.21	-	
Node (TNM)	0.19	-	0.38	-	
Metastasis (TNM)	0.26	-	0.30	-	
Grade	0.67	-	0.99	-	
Oral cavity	0.73	-	0.48	-	
Oropharynx	0.95	-	0.96	-	
Nasopharynx	0.93	-	0.82	-	
Larynx	0.93	-	0.82	-	
Others	0.76	-	0.94	-	
Sex	0.08	-	0.22	-	
ECOG PS	0.41	-	0.24	-	
Tobacco	0.31	-	0.80	-	
Alcohol	0.11	-	0.01	2.34 (1.25-4.35)	
Employed	0.65	-	0.33	-	
Rural	0.68	-	0.09	-	

Tobacco was the only component linked to a greater TD that could be found; however, the correlation was weak (p=0.05, HR: 1.01, 95% CI: 1.0-1.02; -log_2_(p)=4.34). Compared to non-smokers, it was observed that smokers had more stage III (n=16, 21%) and IV (n=43, 55%) HNCs (Appendix 4). To explore further, a negative correlation was found between alcohol consumption and ST (p=0.01, HR: 2.34, 95% CI: 1.25-4.35), despite no impact of alcohol in TD (p=0.11). Also, a longer TIT was related to tumor size (p=0.04, HR: 1.39, 95% CI: 1.01-1.92), but it was associated with lower PT (p=0.04, HR: 0.70, 95% CI: 0.50-0.99). Additionally, there was a favorable correlation between high-grade illness and TIT (p=0.03, HR: 0.57, 95% CI: 0.33-0.96). Tables 3, 4 summarize the variables that were not related to either TD, TIT, PT, and ST.

During the COVID-19 pandemic, 46% (n=64) of diagnoses were associated with a shorter TIT (p=0.04, HR: 0.57, 95% CI: 0.34-0.96). No association was found between the COVID-19 pandemic and TD (p=0.32), PT (p=0.86), ST (p=0.70), and TT (p=0.52). Also, the COVID-19 pandemic didn’t impact survival in this population (p=0.51). Appendix 4 provides further information.

Regarding survival, a higher extension of the primary disease (p=0.01, HR: 2.67, 95% CI: 1.26-5.67), lymph node metastasis (p=0.02, HR: 2.24, 95% CI: 1.12-4.46) were identified as risk factors with a negative impact on OS. However high-grade disease had a positive association with OS (p=0.03, HR: 0.57, 95% CI: 0.33-0.96). No other variables were identified as risk factors (Table 2).

## Discussion

There are several points to highlight in this study. Firstly regarding the study's main objective, TD, TIT, and PT were not associated with OS. The median TD in our study was 4.2 months, which was lower than in some studies [4,17] and higher than in others [10,18] and the median TIT was 1.3 months, longer than some trials [19,20] but lower than the 46-day period associated with an increased risk of mortality [12]. However, due to different definitions of delays and inclusion criteria for malignancies, it is difficult to compare these times with those from other studies.

Secondly, it is important to notice that TD included two times as follows: PT and ST. Regarding PT, it is crucial to note that the majority of patients were symptomatic, which typically signals more symptomatic tumor locations, such as the larynx, or that the illness may be further advanced, which leads people to seek medical attention [18]. The large proportion of patients with advanced clinical stage and the association found between a larger tumor and a lower PT also support that more symptomatic patients tend to be faster when searching for medical help. Conversely, the most prevalent symptoms were odynophagia and dysphonia, which can be misdiagnosed as benign conditions and postpone medical attention [9]. Nevertheless, it is difficult to measure accurately patients' delay because it is based on perception which is highly subjective and can be influenced by many social and cultural factors, although no other associations were found in this trial [17]. Regarding ST, ES was the first option to seek medical help in our sample because in the Portuguese National Health System (NHS) hospital emergency care units are easily accessible and it is challenging to have a primary care consultation. The desire for faster referral to the appropriate specialty or the fear of complications related to more advanced clinical stages may influence ES as people’s first choice. In fact, the diagnostic method may be sped up by ENT's availability in some of the ESs, the possibility of hospitalized patients from the ES to better investigate may have contributed to the high proportion of patients who went to the ES and were diagnosed during hospitalization. However, despite the advantage of being faster, the ER should not be the place to evaluate an HNC patient who doesn’t need urgent intervention. A significant proportion of patients received a primary care consultation, and their median ST was twice as high as that of patients who attended the ES. Therefore, it may also be true that some primary healthcare professionals may fail to recognize some cancer warning signs and symptoms, and that investigation and referral processes might move too slowly [17].

Thirdly, when it concerns treatment delays, other possible causes may be due to the need for more advanced imaging techniques for tumor staging [17], treatment capacity, patients' preferences [21], and administrative issues [11]. As reported in nearly 20% of this sample, patients may also experience a decline in their functional status to the point where they are unable to begin therapy at all, potentially underestimating the median TIT. Also in this trial, the majority of treated patients got multimodal treatment. Also, more aggressive treatments are usually employed to counteract the potential negative consequences of delayed oncological outcomes [13,21]. Multimodality treatments that are required for more advanced stages may also take longer to prepare and schedule [19-21]. But there is controversy in these data [11,18,20] since the relationship between TIT and treatment outcomes is complex [20]. The intensification of the treatment may result in increased morbidity and a worse quality of life.

However, this study did not find a correlation between high-grade toxicity and longer TD, TIT, or survival. Further research is necessary, particularly to measure HNC patients' quality of life, which could serve as a helpful indicator for assessing the effects of treatment when there are delays in diagnosis and treatment [13,20].

Regarding risk factors for diagnosis and treatment delays, despite, the low strength of the association, smoking was found to be a risk factor for a longer TD, but the data on this matter is contradictory. There are studies that have shown that non-smokers had a longer TD compared with smokers [4] and some that showed that only former smokers or only active smokers had the highest risk of delaying searching for medical help [22]. However, there is evidence that advanced stages are correlated with being a smoker, especially a heavy one [18,22]. Alcohol consumption was linked to a longer ST, as previously described in other studies [22]. Potential causes may include the normalization of symptoms and the undervaluing of problems related to illness, which are typical in smokers and drinkers, and contribute to a delay in seeking medical attention and receiving a diagnosis at a later stage of the disease. Also, heavy drinkers tend to miss appointments and try to suppress symptoms with extra alcohol consumption. A longer TIT was linked to a higher T, which represented the burden of advanced disease with comorbidities that typically cause treatment delays. Also, as previously described, usually advanced stages require more complex treatment, resulting in delays in treatment initiation. Surprisingly, a shorter TIT was associated with a higher grade, and a statistically significant positive correlation between OS and grade was also found. One explanation could be related to the biology of these tumors, which are typically rapidly developing and detected at more advanced clinical stages, for example with more lymph nodes metastasis or oligometastatic disease, requiring a prompt course of treatment, and a quick response to that course of treatment which improves TIT [18]. These also serve as an indicator that there are other unknown tumor's biological features that could affect its responsiveness to treatment and consequently OS. Grade is not included in these kinds of studies, but there is one study that addressed tumor growth kinetics with no correlation with survival [20]. Further research is required to elucidate the importance of grade and other biomarkers in this context.

Fourth, COVID-19 didn’t impact TD or survival, but TIT was statistically significantly shorter during this period, as opposed to other trials [23,24]. It is important to note that most clinical activity during this time was focused on COVID-19 patients, with surgery and medical appointments being canceled. In our sample, almost half of the diagnoses were made during COVID-19 period and despite what has been described in other trials, clinical stage and treatment intent were similar in both groups (pre- and post-COVID-19) [24,25]. One plausible explanation is that due to the cessation of most ENT elective surgeries, these patients may have been called faster for surgery, radiotherapy, and/or chemotherapy, regardless of the stage. Although there has been some data suggesting a decrease in cancer diagnoses over this time during this period, our analysis did not support this theory [26].

Lastly, the clinical stage is by far the most important prognostic predictor of this illness [10,13,21,22]. In order to be more accurate, we chose to employ TNM rather than prognostic clinical stage groups. T and N support the negative predictive risk factors of HNC found in our investigation. Probably because of the smaller number of patients in this population, which is indicative of our clinical practice, M was not correlated with OS.

Our study has limitations, and some have already been enlightened before. Nevertheless, being a retrospective trial, it was not possible to control the quality of data based on the clinical records of the consultations. Also, especially in what concerns primary health care and private consultations, data were not available which makes selection bias possible. However prospective data is difficult when studying diagnostic and treatment delays in cancer patients.

## Conclusions

In conclusion, despite the lack of impact of TD or TIT on survival, the Portuguese NHS still needs to shorten the time between first medical consultations, referrals, diagnostic testing, and treatment. Therefore, more awareness is required to recognize the early signs of HNC in the general population as well as healthcare professionals, with special attention on smokers and drinkers. A longer TIT was associated with a larger tumor, supporting multiple hypotheses as follows: treatment delays due to tumor complications, longer preparation times for treatments, longer tumor staging times, and a tendency for some patients to normalize their symptoms. The COVID-19 pandemic was associated with a shorter TIT with no survival impact and more research is needed to ascertain the true impact on HNC patients. Tumor size and nodal metastasis continue to be two of the most significant prognostic variables in HNC patients.

## Appendices

Appendix 1 

**Table 5:** Symptoms of HNC patients. HNC: head and neck cancer; n: number of patients

Symptoms	n (%)
Asymptomatic	4 (2.9)
Only one symptom	73 (52.5)
Dysphonia	52 (37.4)
Odynophagia	35 (25.2)
Neck Tumefaction	31 (23.5)
Dysphagia	17 (12.2)
Head tumefaction	9 (6.8)
Dyspnea	6 (4.3)
Dry cough	4 (2.9)
Nasal obstruction	4 (2.9)
Mouth ulcer	3 (2.2)
Foreign body sensation	3 (2.2)
Epistaxis	3 (2.2)
Headache	2 (1.4)
Toothache	2 (1.4)
Otalgia	2 (1.4)
Mouth pain	2 (1.4)
Facial paresis	2 (1.4)
Hypoacusis	2 (1.4)
Nasal congestion	1 (0.7)
Weight loss	1 (0.7)
Anorexia	1 (0.7)
Asthenia	1 (0.7)
Neck pain	1 (0.7)
Rhinorrhea	1 (0.7)
Nose injury	1 (0.7)
Hematic losses (other than epistaxis)	1 (0.7)
Productive cough	1 (0.7)
Decreased visual acuity	1 (0.7)
Dizziness	1 (0.7)
Xeropthalmia	1 (0.7)

Appendix 2 

**Table 6:** Characteristics regarding diagnosis and treatment. RT: radiotherapy; n: number of patients

Factors	n (%)
First medical contact	
Emergency room	59 (42)
Primary health care	52 (37)
ENT private consultation	16 (12)
Dentist	2 (1)
Others	6 (4)
Missing	4 (4)
Place of the first specialty visit	
Consultation	76 (55)
Emergency room	63 (45)
First specialty contact	
ENT	121 (87)
Surgery	14 (10)
Internal medicine	4 (3)
Medical oncology	1 (1)
Missing	1(1)
Surgery	
Internal medicine	14 (10)
Medical oncology	4 (3)
Missing	1 (1)
Diagnostic techniques	
Incisional tumor biopsy	96 (69)
Excisional tumor biopsy	35 (25)
Cytology of the adenopathy	8 (6)
Excisional adenopathy biopsy	2 (1)
Place of diagnosis	
ER	31 (22)
Consultation	34 (25)
Hospitalization	72 (52)
Missing	1(0.7)
No treatment	25 (18)
Treatment	114 (82)
Curative intent	110 (88)
Surgery alone	26 (23)
Surgery plus RT	21 (18)
Surgery plus ChT/RT	10 (9)
Radical RT	4 (4)
ChT/RT	25 (22)
ChT/RT plus adjuvant chemotherapy (ChT)	4 (4)
Induction ChT plus ChT/RT	6 (5)
Induction ChT plus RT	2 (2)
Palliative intent	12 (11)
Palliative RT	4 (4)
Systemic palliative treatment	8 (7)

Appendix 3 

**Table 7:** Toxicity regarding patient’s treatment. n: number of patients

Toxicity	Grade 1-2	Grade 3-4	Delay	Dose reduction	Discontinuation
	n (%)				
Anemia	55 (48.2)	4 (3.5)	6 (5.3)	-	-
Neutropenia	21 (18.4)	8 (7.0)	14 (12.3)	1 (0.9)	-
Trombocitopenia	10 (8.8)	1 (0.9)	-	-	-
Mucositis	45 (39.5)	8 (7.0)	6 (5.7)	1 (0.9)	2 (1.8)
Xerostomia	29 (25.4)	-	-	-	-
Acute renal failure	6 (5.7)	-	1 (0.9)	1 (0.9)	-
Peripheral neuropathy	2 (1.8)	-	-	-	-
Gastrointestinal	33 (28.9)	12 (10.5)	11 (9.6)	-	3 (2.6)
Skin	53 (46.5)	6 (5.7)	4 (3.5)	1 (0.9)	-
Infections	20 (17.5)	1 (0.9)	11 (9.6)	-	1 (0.9)
Thromboembolic events	-	1 (0.9)	1 (0.9)	-	1 (0.9)
Cardiac toxicity	-	1 (0.9)	1 (0.9)	-	1 (0.9)
Convulsive crisis	1 (0.9)	-	1 (0.9)	-	1 (0.9)

Appendix 4 

**Table 8:** Characteristics of smoker and former/non-smokers patients and patients diagnosed before and after COVID-19 pandemic. COVID-19: coronavirus disease 2019; n: number of patients

Stage	Actively smokers	Former smokers/non-smokers	COVID-19	Non-COVID-19
	n (%)			
T1	16 (21)	10 (14)	9 (14)	16 (22)
T2	11 (14)	13 (18)	14 (22)	10 (14)
T3	26 (33)	16 (22)	17 (27)	24 (32)
T4	25 (32)	18 (25)	22 (34)	23 (31)
N0	32 (41	31 (43)	31 (48)	16 (22)
N1	9 (12)	6 (8)	9 (14)	10 (14)
N2	22 (28)	18 (25)	14 (22)	24 (32)
N3	15 (19)	4 (6)	10 (16)	23 (31)
M1	6 (8)	3 (4)	4 (6)	5 (7)
I	14(18)	10 (13)	9 (14)	14 (20)
II	6 (8)	9 (012)	10 (16)	5 (7)
III	16 (21)	11 (15)	12 (19)	14 (22)
IV	43 (55)	29 (40)	33 (52)	41 (57)
